# Mediterranean diet and insulin sensitivity, lipid profile and blood pressure levels, in overweight and obese people; The Attica study

**DOI:** 10.1186/1476-511X-6-22

**Published:** 2007-09-19

**Authors:** Natalia Tzima, Christos Pitsavos, Demosthenes B Panagiotakos, John Skoumas, Antonis Zampelas, Christina Chrysohoou, Christodoulos Stefanadis

**Affiliations:** 1 Department of Nutrition and Dietetics, Harokopio University, Athens, Greece; 2 First Cardiology Clinic, School of Medicine, University of Athens, Athens, Greece; 3 46 Paleon Polemiston St. Glyfada, Attica, 166 74, Greece

## Abstract

**Background:**

We aimed to investigate if overweight and obese adults "close" to Mediterranean diet present better insulin, lipids profile and better pressure levels, compared to individuals close to a more Westernized diet.

**Methods:**

The ATTICA study is a population-based cohort that has randomly enrolled 3042 adult men and women, stratified by age – gender, from the greater area of Athens, during 2001–2002. Of them, in this work were have studied 1762 participants with excess body weight, meaning overweight (BMI: 25–29.9 kg/m^2^) and obese (BMI>30 kg/m^2^). 1064 were men and 698 women (20–89 years old). Adherence to Mediterranean diet was assessed through a diet-score that was based on a validated food-frequency questionnaire. Blood pressure was measured and also fasting glucose, insulin and blood lipids. Insulin sensitivity was also assessed by the homeostasis model assessment (HOMA) approach (glucose × insulin/22.5).

**Results:**

Individuals with excess bodyweight in the highest tertile of diet score, were more insulin sensitive than those in the lowest tertile (11.4% lower HOMA, p = 0.06), had 13% lower levels of total cholesterol (p = 0.001) and 3 mmHg decrease of systolic blood pressure levels (p < 0.001), when adjusted for age, sex and BMI. Multivariate analysis after taking into account several confounders demonstrated that insulin sensitivity, total cholesterol and systolic blood pressure were independently but only modestly correlated with Mediterranean diet in people with excess bodyweight.

**Conclusion:**

Adherence to Mediterranean diet is modeslty associated with a better insulin sensitivity, lower levels of total cholesterol and lower levels of systolic blood pressure in overweight and obese subjects. This may suggest that compared to general population, the beneficial effect of this diet in cardiovascular system of excess body weight people is limited.

## Background

Obesity is expanding worldwide and is an important risk factor associated with coronary heart disease [1]. The fraction of population with excess bodyweight is rapidly increasing also in Europe. According to International Obesity Task Force (IOTF), Greece shows the highest prevalence for both overweight and obesity in women (74%) and the second in men (72%), among different European countries [2]. More recent data from Attica study show that prevalence of overweight and obesity were 53% and 20% in men and 31% and 15% in women respectively [3]. Even though, the risk for coronary heart events is relatively low among Greeks [4].

In reality, the exact mechanism by which obesity promotes atherosclerosis is not fully understood. The interrelationship between obesity and insulin resistance, dyslipidemia and hypertension may contribute to cardiovascular risk, but is not known to what extent [5,6]. Many studies have revealed, for instance, that insulin resistance is not a necessary component of obesity [7]. Furthermore, it becomes more and more evident the existence of an entity, named as "uncomplicated obesity" [8]. So, excess bodyweight subjects can be divided to normolipidemic, insulin sensitive, normotensives ones and to those with complications as insulin resistance, dyslipidemia and hypertension. Identifying these two subgroups is not without difficulties, but is crucial, as they obviously demand different level of aggressiveness in interventions and prevention measures [9].

Diet must be considered as an essential aspect of prevention, since its impact on the development of coronary heart disease is being demonstrated by many studies. [10,11] Especially, the atheroprotective role of Mediterranean diet is not questioned [12,13]. Beyond that, the Attica study has confirmed that adherence to Mediterranean diet is linked with less obesity [14]. Nevertheless, it is not yet investigated if Mediterranean diet is correlated with fewer complications in people already of an excess weight.

Adherence to Mediterranean diet has already been correlated with better glycemic indices, with a more attractive lipid profile and with lower blood pressure levels in general population [15-17]. Some studies have also investigated these relationships in people with metabolic syndrome or in elderly people but few have focused on the excess body weight population, already in high cardiovascular risk [18-20]. Hence, the aim of the present work was to evaluate whether overweight and obese adults attached to a Mediterranean dietary pattern have lesser co-factors for cardiovascular disease (i.e., better insulin and lipids profile as well as lower blood pressure levels), compared to individuals close to a more Westernized diet.

## Methods

### Population of the study

The "ATTICA" study is a health and nutrition survey, which is being carried out in the province of Attica (including 78% urban and 22% rural areas), where Athens, is a major metropolis. The sampling was random, multistage and it was based on the age – sex distribution of the province of Attica provided by the National Statistical Service (census of 2001). The sampling anticipated enrolling only people without any clinical history of cardiovascular disease, or any other atherosclerotic disease, as well as chronic viral infections. Moreover, participants did not have cold or flu, acute respiratory infection, dental problems or any type of surgery during the past weeks. Also, all people living in institutions were excluded from the study. From May 2001 to December 2002, 4056 inhabitants from the above area were randomly selected to enroll into the study. Of them, 3042 agreed to participate (75% participation rate). The selected sample can be considered as representative since there were only minor, insignificant, differences in sex and age distribution between the study population and the target population.

In this work, we focused on excess bodyweight people, i.e. obese with a BMI>30 kg/m^2^, (n = 536) and overweight with a BMI: 25–29.9 kg/m^2 ^(n = 1226), according to WHO criteria [21]; 1064 of the participants were men (20–87 years old) and 698 were women (20–89 years old). Moreover, 63.3% of them were physically inactive, 42.7% current smokers, 43.1% dyslipidemic, while the prevalence of hypertension and type 2 diabetes mellitus in our study population was 39.9% and 9.7%, respectively. The characteristics of our participants have already been presented in more details, elsewhere [3]. All participants were interviewed by trained personnel (cardiologists, dieticians and nurses) who used a standard questionnaire that evaluated lifestyle habits and various socio-demographic, clinical and biological characteristics. Power analysis showed that the number of enrolled participants is adequate to evaluate standardised differences of the investigated biological parameters greater than 0.5, achieving statistical power greater than 0.80 at 5% probability level (P-value).

### Dietary assessment

Consumption of non-refined cereals and products, vegetables, legumes, fruits, olive oil, dairy products, fish, pulses, nuts, potatoes, eggs, sweets, poultry, red meat and meat products were measured as an average per week during the past year through a validated food – frequency questionnaire that was kindly provided by the department of Epidemiology, of Athens Medical School [22]. The frequency of consumption was then quantified approximately in terms of the number of times a month a food was consumed. Alcohol consumption was measured by daily ethanol intake, in wineglasses (100 ml and 12% ethanol concentration).

The Mediterranean dietary pattern consists of: (a) daily consumption: of non refined cereals and products (whole grain bread, pasta, brown rice, etc), vegetables (2 – 3 servings/day), fruits (6 servings/day), olive oil (as the main added lipid) and dairy products (1 – 2 servings/day), (b) weekly consumption: of fish (4–5 servings/week), poultry (3 – 4 servings/week), olives, pulses, and nuts (3 servings/week), potatoes, eggs and sweets (3 – 4 servings/week) and monthly consumption: of red meat and meat products (4 – 5 servings/month). It is, also, characterized by moderate consumption of wine (1 – 2 wineglasses/day) and high monounsaturated: saturated fat ratio (> 2). A Harvard-led group suggested this dietary pyramid with substantial input from Greek scientists [23]. Based on this dietary pyramid we calculated a special diet score that assessed adherence to Mediterranean diet. In particular, for the consumption of food items that are close to this dietary pattern we assigned score 0 for rare or no consumption, 1 for 1 to 4 times/month, 2 for 5 to 8 times, 3 for 9 to 12 times/month, 4 for 13 to 18 times/month and 5 for almost daily consumption. On the other hand, for the consumption of foods that are away from this traditional diet, like meat and meat products, we assigned the opposite scores (i.e. 0 for almost daily consumption to 5 for rare or no consumption). For alcohol, we assigned score 5 for consumption of less than 3 wineglasses/day and, progressively, score 0 for consumption of more than 7 wineglasses/day. Thus, the range of the diet score was between 0 – 55. Higher values of the suggested dietary score indicate greater adherence to the traditional Mediterranean diet (i.e. which is also characterized by moderate consumption of fat and high monounsaturated: saturated fat ratio); while in the present work we classified our sample into the commonly used tertiles of the diet score as done in previous works of the ATTICA Study [24].

### Biochemical measurements

The blood samples were collected from the antecubital vein between 8 to 10 a.m., in a sitting position after 12 hours of fasting and avoiding of alcohol. Serum for the measurement of these lipids was harvested immediately after admission. The biochemical evaluation was carried out in the same laboratory that followed the criteria of the World Health Organization Lipid Reference Laboratories. Serum total cholesterol, HDL-cholesterol and triglycerides were measured using chromatographic enzymic method in a Technicon automatic analyser RA-1000 (Dade Behring, Marburg, Germany). LDL cholesterol calculated using the Friedewald formulae: {total cholesterol} – {HDL cholesterol} – 1/5 (triglycerides). An internal quality control was in place for assessing the validity of cholesterol, triglyceride and HDL methods. The intra and inter-assay coefficients of variation of cholesterol levels did not exceed 9%, of triglycerides 4% and of HDL 4%. Serum insulin concentrations were assayed by means of radioimmunoassay (RIA100, Pharmacia Co., Erlangen, Germany). Insulin sensitivity was assessed by the calculation of the homeostasis model assessment (HOMA) approach (glucose in mmol/l × insulin in μU/mL/22.5).

### Demographic, clinical and lifestyle characteristics

The study's questionnaire also included demographic characteristics like age, gender, financial status, and education level. Information about smoking habits was collected using a standardized questionnaire developed for the Study. Current smokers were defined as those who smoked at least one cigarette per day. Never smokers those who have never tried a cigarette in their life and former smokers were defined as those who had stopped smoking more than one year previously. For the multivariate statistical analyses cigarette smoking was quantified in pack-years (cigarette packs per day X years of smoking), adjusted for a nicotine content of 0.8 mg/cigarette.

For the ascertainment of physical activity status, we used the short version of the International Physical Activity Questionnaire (IPAQ, [25]), as an index of weekly energy expenditure using frequency (times per week), duration (in minutes per time) and intensity of sports or other habits related physical activity. Particularly, intensity was gradated in qualitative terms such as: light (expended calories < 4 Kcal/min, i.e. walking slowly, cycling stationary, light stretching etc.), moderate (expended calories 4–7 Kcal/min, i.e. walking briskly, cycling outdoor, swimming moderate effort etc.) and high (expended calories >7 Kcal/min, i.e. walking briskly uphill, long distance running, cycling fast or racing, swimming fast crawl etc.). Participants who did not report any physical activities were defined as sedentary. For the rest of the participants we calculated a combined score by multiplying the weekly frequency, duration and intensity of physical activity.

Arterial blood pressure was measured three times at the end of the physical examination with subject in a sitting position. Patients whose average blood pressure levels were greater or equal to 140/90 mm Hg or were under antihypertensive medication were classified as hypertensives. Hypercholesterolemia was defined as total serum cholesterol levels greater than 220 mg/dl or the use of lipid-lowering agents and diabetes mellitus as a blood sugar > 125 mg/dl or the use of antidiabetic medication [26]. The Ethical Committee of our Department has approved the study.

### Statistical analysis

Continuous variables are presented as mean values ± one standard deviation, while qualitative variables are presented as absolute and relative frequencies. Comparisons between normally distributed continuous variables and categorical were performed by the calculation of Student's t-test and one-way or multi-way Analysis of Co-Variance (MANCOVA), after testing for equality of variances (homoscedacity). Shapiro – Wilk test was used to assess normality of the distributions. Mean values for glucose, insulin, HOMA, blood lipids, systolic and diastolic blood pressure, in the different tertiles of Mediterranean diet score were tested using generalised linear models for fixed effects, after adjusting for age, sex, BMI, hypertension, diabetes, special treatment, physical activity, W/H, and smoking. All reported *P*-values are based on two-sided tests and compared to a significance level of 5%. SPSS 13.0 software (SPSS Inc. 2003, Chicago, Illinois, USA) was used for all the statistical calculations.

## Results

Table 1 illustrates various characteristics of the participants by their Mediterranean diet status. As we progress from a diet score "away" (1^st ^tertile of diet score) towards a score "very close" to the Mediterranean type of diet, we observe that excess bodyweight people are younger, more educated and more active and in greater portion females and smokers. Furthermore, the prevalence of obesity and central obesity decreases.

**Table 1:** Various characteristics of the overweight/obese participants according to Mediterranean diet score.

	Mediterranean Diet Score			
	1^st ^tertile <24	2^nd ^tertile 24–27	3^rd ^tertile >27	P

*N*	*901*	*722*	*139*	
Age (years)	55 ± 13	46 ± 11	35 ± 10	< 0.001
Males%	76	58	16	< 0.001
Years of school	11 ± 4	12 ± 4	13 ± 3	< 0.001
Current smoking%	38	46	45	0.002
Physical activity%	39	38	44	0.014
Waist (cm)	101.9 ± 12.7	92.2 ± 11.2	77.7 ± 10.6	< 0.001
Waist – to – hip	0.93 ± 0.09	0.87 ± 0.09	0.78 ± 0.08	< 0.001
BMI (Kg/m^2^)	29.6 ± 4.4	26.6 ± 2.8	22.5 ± 2.6	< 0.001

Table 2 presents fasting glycemic control indices, blood lipids and blood pressure levels in people with excess bodyweight along with the diet score, after adjusting for age, sex and BMI. We observe that compared to first tertile, obese and overweight people in the third tertile of diet score have 4.4% lower insulin levels (p = 0.06), 6% lower glucose levels (p = 0.08), 11.4% lower HOMA levels (p = 0.06), 13% lower cholesterol (p = 0.001). Compared to those with a low diet score, those with a score in the third tertile, had 3 mmHg lower systolic blood pressure levels (p < 0.001) and 7 mmHg lower diastolic pressure levels (p = 0.02). Participants very close to Mediterranean diet had lower levels of triglycerides compared to those away from this pattern (129 vs. 146) but not in the level of statistical significance. Differences in the mean values of HDL were also not statistically significant.

**Table 2:** Age, sex and body mass index adjusted mean values of glycemic control indices, blood lipids and arterial blood pressure levels.

	Mediterranean Diet Score			
	1^st ^tertile	2^nd ^tertile	3^rd ^tertile	P

*N*	*901*	*722*	*139*	
Insulin (μU/mL)	13.7 ± 0.1	13.3 ± 0.1	13.1 ± 0.2	0.06
Blood glucose (mg/dl)	98 ± 1	94 ± 1	92 ± 2	0.08
HOMA-R	3.5 ± 0.1	3.2 ± 0.1	3.1 ± 0.1	0.06
Total cholesterol (mg/dl)	210 ± 2	201 ± 2	183 ± 4	0.001
HDL-C (mg/dl)	45.3 ± 0.6	46.1 ± 0.6	47.6 ± 0.4	0.15
Triglycerides (mg/dl)	146 ± 4	144 ± 4	129 ± 11	0.18
Systolic BP (mmHg)	128 ± 1	127 ± 1	125 ± 1	< 0.001
Diastolic BP (mmHg)	84 ± 1	83 ± 1	77 ± 1	0.02

As Table 3 illustrates, we further investigated the relation of glycemic control indices, blood lipids and blood pressure levels with diet score in models of multiple linear regression analysis, after taking into account additional variables, like hypertension, diabetes, special treatment, physical activity, waist to hip ratio and smoking. We observed that Mediterranean diet score was still in correlation with HOMA (p = 0.09), blood cholesterol (p = 0.05) and slightly with systolic blood pressure (b-coefficient: -0.09 ± 0.1, p = 0.08). In the models of multiple regression analysis, insulin and diastolic blood pressure levels were not found to be independently correlated with diet score.

**Table 3:** Results from multi-adjusted analysis for the evaluation of the Mediterranean diet (independent) on blood lipids, glycemic control indices and blood pressure levels.

Dependent variable	b-coefficient ± SE*	R^2 ^with/without the diet score in the model	p
*Model 1: Insulin (μU/mL)*	-0.17 ± 0.1	0.22/0.20	0.27
*Model 2: HOMA-R*	-0.26 ± 0.2	0.38/0.33	0.09
*Model 3: Total cholesterol (mg/dl)*	-0.30 ± 0.1	0.62/0.54	0.05
*Model 4: HDL-C (mg/dl)*	-0.04 ± 0.1	0.08/0.07	0.96
*Model 5: Triglycerides (mg/dl)*	0.55 ± 0.8	0.14/0.12	0.51
*Model 6: Systolic BP (mmHg)*	-0.09 ± 0.1	0.51/0.47	0.08
*Model 7: Diastolic BP (mmHg)*	0.08 ± 0.2	0.10/0.08	0.51

*Independent variables also included in the model are: age, sex, co-morbidities (hypertension, diabetes, dyslipidemia) and special treatment (antidiabetic drugs for model 1, 2, lipids lowering agents for models 3, 4, 5, antihypertensive drugs for models 6, 7), physical activity, years of school, waist-to-hip ratio, cigarette smoking and BMI.

## Discussion

In the present work we revealed that in overweight and obese people, a greater adherence to Mediterranean diet is only modestly associated with higher insulin sensitivity, better lipid profile and lower blood pressure levels. ATTICA study has already shown that subjects that are "close" to Mediterranean diet have better fasting indices of glucose homeostasis and less insulin resistance compared with subjects that follow a more westernized diet [27]. Compared to these findings from general population, our results that focused on excess weight population show that an increment in the diet score is associated with moderately reduced HOMA-R, after adjustment for age, sex, hypertension, diabetes, dyslipidemia, physical activity, waist to hip ratio, smoking and BMI. Although the relationship between Mediterranean diet and insulin sensitivity has been investigated in healthy people, it has not specifically investigated in overweight and obese people [28]. Nevertheless, Esposito et al. confirmed this relationship in people with obesity-related metabolic syndrome [18]. In contrary to HOMA, insulin levels were not correlated with diet score when several variables were taking into account. Hence, we must assume that mostly insulin sensitivity and not the secretory capacity of pancreatic cells, as estimated by insulin levels, is independently correlated with diet score in excess weight people.

Our study has also shown that in overweight/obese population, adherence to Mediterranean diet is related in a limited way (p = 0.05) with a decrease in total cholesterol, when taking into account different variables (diabetes, hypertension etc) in a multivariate analysis. So, compared to the general population, the relation of Mediterranean diet with lipid profile in excess body weight people, is less important and powerful [29,30]. Although it is not aroused a specific interest for the significance of this relationship in people with excess but stable weight, there are some intervention studies which explore this correlation in obese and overweight people who attempt to lose weight. Pelkman et al., have found that the adoption of a weight loss diet, based on a moderate consumption of monounsaturated fat (a constitutional component of Mediterranean diet) has a favourable effect on the serum lipid profile of overweight and obese people [31]. As our results have not shown an independent correlation of HDL and triglycerides with diet score, we must assume that the poor correlation of total cholesterol is due mostly to LDL cholesterol.

Moreover, our study reveals that increase in the diet score is associated with a statistically significant – but of minor clinical importance-decrease of systolic blood pressure in overweight/obese population, after adjusting for several potential confounders. Hence, the aforementioned association in overweight/obese population is different than in general population, as previous data from ATTICA study have revealed. According to these findings, the consumption of Mediterranean diet is associated with lower prevalence and better control of high blood pressure levels and this is in agreement with the Lyon Diet Heart study and other studies. [17,30,32]. We did not find an independent association of diastolic blood pressure with Mediterranean diet in overweight and obese people, when we analyze further with multiple regression models. This maybe due to the small variation of diastolic pressure or to the fact that the diastolic pressure is correlated to obesity per se rather than any type of diet.

The fact that excess body weight is correlated with higher levels of insulin resistance, blood lipids and blood pressure levels and also with a higher prevalence of hypertension, diabetes and hyperlipidemia may partially explain the modest impact of Mediterranean diet in this case [5]. Moreover, the protective role of Mediterranean diet may be weaker as it cannot counteract the effects of obesity-related increased inflammatory adipokines which promote and maintain the abovementioned cardiovascular risk factors. Furthermore, according to previous data from Attica study, overweight and obese men and women have a lesser diet score compared with those with normal weight and this may further elucidate the results of our study [3].

## Limitations

This is a cross-sectional study, so it could not establish causal relationships but only states hypotheses about the link between different parameters and Mediterranean diet in people with excess body weight. Therefore, the possible modest impact of Mediterranean diet on lipid profile, HOMA levels and blood pressure levels in obese and overweight people should be further investigated by randomised clinical trials. Another limitation of our study is concerning a possible overestimation of blood pressure due to the "white coat effect". Based on previous observational studies, average blood pressure of another visit may give lower values than our initial visit, due to regression to the mean or the familiarization of the participants to the clinic setting. However, our methodology is similar to those of other cross – sectional surveys in Europe and the US, and therefore the results are comparable [33]. In this study, we did not use the gold standard, the euglycaemic-hyperinsulinaemic clamp for the assessment of insulin sensitivity. However, other investigators reported that HOMA is strongly related to clamp-measured insulin resistance in both non-diabetic and diabetic subjects [34]. The fact that a possible vast difference in distribution of unknown or unmeasured variables cannot be addressed must be considered as another limitation of our study. Finally, misreporting of food items consumed could influence the calculation of the diet score and bias the results for the data analysis.

## Conclusion

To the best of our knowledge this is the first cross-sectional study that shows that this high risk group of overweight and obese people have a limited profit regarding cardiovascular risk factors, if they are attached to the Mediterranean dietary pattern. However, as Mediterranean diet is acceptable by most of the people for long term, its adoption could play some role in preventing or delaying coronary disease in overweight and obese people that are already at risk, but further investigation is needed in this field.

## Competing interests

The author(s) declare that they have no competing interests.

## Authors' contributions

NT conceived the study, wrote the manuscript and interpreted the results, CP designed the study and drafted the manuscript, DBP designed the study and assisted in the statistical analysis, JS drafted the manuscript, AZ drafted the manuscript, CC designed the study and critically reviewed the manuscript, CS critically reviewed the manuscript. All authors read and approved the final manuscript.
